# Cytoadherence Properties of *Plasmodium knowlesi*-Infected Erythrocytes

**DOI:** 10.3389/fmicb.2021.804417

**Published:** 2022-01-05

**Authors:** Wenn-Chyau Lee, Shahhaziq Shahari, Samantha Yee Teng Nguee, Yee-Ling Lau, Laurent Rénia

**Affiliations:** ^1^Department of Parasitology, Faculty of Medicine, Universiti Malaya, Kuala Lumpur, Malaysia; ^2^A*STAR Infectious Diseases Labs, Agency for Science, Technology and Research (A*STAR), Singapore, Singapore; ^3^Department of Microbiology & Immunology, Yong Loo Lin School of Medicine, National University of Singapore, Singapore, Singapore; ^4^Lee Kong Chian School of Medicine, Nanyang Technological University, Singapore, Singapore; ^5^School of Biological Sciences, Nanyang Technological University, Singapore, Singapore

**Keywords:** *Plasmodium knowlesi*, cytoadherence, endothelial, rosette, receptors

## Abstract

*Plasmodium knowlesi* is responsible for zoonotic malaria infections that are potentially fatal. While the severe pathology of falciparum malaria is associated with cytoadherence phenomena by *Plasmodium falciparum*-infected erythrocytes (IRBC), information regarding cytoadherence properties of *P. knowlesi*-IRBC remained scarce. Here, we characterized the cytoadherence properties of RBC infected with the laboratory-adapted *P. knowlesi* A1-H.1 strain. We found that late-stage IRBC formed rosettes in a human serum-dependent manner, and rosettes hampered IRBC phagocytosis. IRBC did not adhere much to unexposed (unstimulated) human endothelial cell lines derived from the brain (hCMEC/D3), lungs (HPMEC), and kidneys (HRGEC). However, after being “primed” with *P. knowlesi* culture supernatant, the IRBC-endothelial cytoadherence rate increased in HPMEC and HRGEC, but not in hCMEC/D3 cells. Both endothelial cytoadherence and rosetting phenomena were abrogated by treatment of *P. knowlesi*-IRBC with trypsin. We also found that different receptors were involved in IRBC cytoadherence to different types of endothelial cells. Although some of the host receptors were shared by both *P. falciparum-* and *P. knowlesi-*IRBC, the availability of glycoconjugates on the receptors might influence the capacity of *P. knowlesi*-IRBC to cytoadhere to these receptors.

## Introduction

Malaria is still one of the significant healthcare burdens in many tropical and subtropical countries. The efforts invested to eradicate malaria have witnessed encouraging progress as the numbers of malaria-associated deaths decrease year by year. Nevertheless, the malaria elimination program is challenged by the emergence and persistent occurrence of zoonotic malaria in Southeast Asia and South America (Luchavez et al., 2008; Figtree et al., 2010; Brasil et al., 2017; Grigg et al., 2017; Anstey and Grigg, 2019). As wild primates serve as the reservoirs of zoonotic malaria parasites, infections will be difficult, if not impossible to be completely eradicated from the human populations of these affected areas. A few species of simian malaria parasites are transmissible to humans (Coatney et al., 1966; Ta et al., 2014; Hartmeyer et al., 2019). However, majority of the reported symptomatic natural zoonotic malaria cases in Southeast Asia are attributed to *P. knowlesi* (Figtree et al., 2010; Lee et al., 2013a; Siner et al., 2017; Chin et al., 2020), making it the fifth species of medically important malaria parasites (White, 2008). With its much shorter erythrocytic cycle (∼24 h), *P. knowlesi* can propagate rapidly, causing quick progression of pathogenesis to potentially fatal outcomes, which manifest in several forms, such as acute respiratory distress syndrome (ARDS) and acute renal failure (ARF) (Daneshvar et al., 2009; William et al., 2011; Singh and Daneshvar, 2013).

Our current understandings on severe malaria pathogenesis are mostly based on the in-depth studies conducted on *Plasmodium falciparum*, the most fatal human malaria parasite. This is also the species that has an established *in vitro* cultivation system for decades (Trager and Jensen, 1976), which has facilitated biological and immunological studies. The severe pathogenesis in falciparum malaria is thought to be associated with the ability of *P. falciparum*-infected erythrocytes (IRBC) to stably adhere to endothelial cells (a phenomenon known as IRBC–endothelial cytoadherence) and uninfected erythrocytes (URBC) (a phenomenon called rosetting) (Craig et al., 2012; Lee et al., 2019). These phenomena disrupt blood flow and activate the endothelial cells, resulting in vascular leakage and injury, which are the hallmarks of severe malaria pathology. Cerebral malaria is one of the most well-studied falciparum malaria-associated complications. It is not known if the knowledge obtained from *P. falciparum* studies can be extrapolated to other parasites, such as *P. knowlesi*. Contrary to *P. falciparum*, neurological complications are rarely seen in severe knowlesi malaria. However, other severe pathologies affecting the lungs and kidneys are frequently observed (Cox-Singh et al., 2010; Kantele and Jokiranta, 2011; William et al., 2011).

Little information is available regarding the cytoadherence properties of *P. knowlesi*-IRBC. One earlier study reported the binding of *P. knowlesi*-IRBC to recombinant human ICAM-1 and VCAM proteins coated on Petri dishes (Fatih et al., 2012). Apart from that, several rheological studies have reported increased rigidity of *P. knowlesi*-IRBC (Barber et al., 2018b; Liu et al., 2019), which may affect cytoadherence properties of the IRBC. Thus, the actual binding interactions of *P. knowlesi*-IRBC with any of the host cells remained to be characterized to clearly understand the pathobiology of knowlesi malaria. Here, we characterized the cytoadherence properties of *P. knowlesi* A1-H.1, the reference strain that can be propagated *in vitro* with human RBC (Moon et al., 2013).

## Materials and Methods

### Materials Used

Information of materials used is available in Supplementary Table 1.

### Study Approval

Experiments were conducted using the ethical guideline NMRR-17-1718-35558 approved by the Medical Research and Ethics Committee (MREC), Ministry of Health, Malaysia. Usage of human blood samples in the experiments were approved by the University of Malaya Medical Centre Medical Ethics Committee (Ref. MEC No. 817.18).

### Parasite and Endothelial Cell Cultures

Parasite (*P. knowlesi* A1-H.1) cultures were maintained at 37°C, humidity >90%, and gas mixtures of 5% CO_2_, 5% O_2_ (henceforth, the “*in vitro* cultivation conditions”). The parasite cultures were constantly maintained at 5% hematocrit, 5% parasitemia with human RBC of group “O” and RPMI-1640 media enriched with AlbuMAX II and 10% (v/v) horse serum [used at the beginning of the study, as described elsewhere (Moon et al., 2013), subsequently adapted to RPMI-1640 media enriched with 20% (v/v) heat-inactivated human AB sera]. After the parasite cultures were maintained with human serum-enriched medium, the parasite culture supernatant was collected during medium changes. The collected culture supernatant was centrifuged at 1,000 × *g* for 10 min to sediment cellular debris. These supernatant aliquots were stored at −80°C for subsequent use. For each batch of culture supernatant collected, an aliquot of batch matching, unused parasite culture medium was also stored for later use in experiments. Endothelial cell lines were cultured on rat tail collagen (RTC)-coated surface [100 μg/ml of RTC in 0.02 N acetic acid was coated on the cell culture surface (culture flask or slides) for 4 h at 37°C, followed by removal of the RTC solution and rinsed with 1× PBS] with endothelial cell medium (ECM) kit that was prepared according to instructions provided by the manufacturer. Human monocytic THP-1 and Chinese hamster ovarian (CHO) cell lines were cultured in 10% fetal bovine serum (FBS)-enriched RPMI-1640. All parasite and cell line cultures were *Mycoplasma*-free as ascertained using the MycoAlert™ Plus *Mycoplasma* detection kit.

### Rosetting Assay

Rosetting assays were conducted on a daily basis using the Giemsa-stained wet mount technique as described elsewhere (Lee et al., 2013b; Lee and Rénia, 2020). Briefly, the parasite culture suspension was stained subvitally with Giemsa (5% v/v) prior to wet mounting on a glass slide with a glass coverslip for immediate examination using light microscope under oil immersion (×1,000) magnification. We adapted the parasite culture to 20% human AB serum-enriched medium and repeated the rosetting assay 24 h l (denoted as the first cycle). The rosetting assay was repeated every cycle [twice, to examine rosette availability at early (ring) stage and late (trophozoite–schizont) stage] for 14 consecutive cycles to follow the trend of rosetting rate, which is the percentage of IRBC that formed rosettes (by counting 200 IRBC). Five biological replicates were performed (five flasks of parasite cultures derived from different batches of cultures that were previously cultivated with human serum-free media but adapted to human serum-enriched media separately).

In a separate experiment, late-stage IRBC were purified with a magnetic activated cell sorter (MACS). The purified IRBCs were divided into three groups. Two groups were treated with different concentrations of trypsin (final concentrations of 10 and 1 mg/ml, respectively). The third group served as untreated control. The enzyme treatment was conducted for 5 min at 37°C. Subsequently, the enzymatic reaction was stopped, and the treated packed IRBCs were washed with culture medium three times. The non-enzymatic-treated URBCs were added to the packed IRBCs and suspended with human serum-enriched culture medium to constitute parasite culture suspension (5% parasitemia, 5% hematocrit). Culture suspensions were incubated at culture conditions for 1 h prior to rosetting assessment. Ten biological replicates were conducted.

Parasite cultures with stable rosetting phenotypes were prepared. Culture suspensions were centrifuged, and packed cells were divided into two groups after three rounds of washings with 1× PBS. One group was suspended with 20% human serum-enriched medium, whereas another group was suspended with human serum-free medium (enriched with AlbuMAX II + horse serum). The cell suspensions were incubated under culture conditions for 1 h, prior to rosetting rate assessment. Subsequently, IRBC phagocytosis evaluation was performed as described elsewhere (Lee et al., 2020). Briefly, the human monocytic THP-1 cells were added (ratio of THP-1: RBC = 1:10,000) to the parasite cultures (one group in human serum-enriched medium, and the other one in the human serum-free medium) and incubated under *in vitro* cultivation conditions for 1 h. Subsequently, IRBC phagocytosis rate (percentage of phagocytes with IRBC engulfment; mere phagocyte contact with IRBC without evident formation of pseudopods were excluded) was determined with wet mount by recruiting 1000 THP-1. Simultaneously, the rosetting rates of the wet mounts were evaluated. Ten biological replicates were conducted.

### Infected Erythrocytes–Endothelial Cytoadherence Assay

Three human endothelial cell lines were used, namely, human cerebral microvascular endothelial cells (hCMEC/d3), human pulmonary microvascular endothelial cells (HPMEC), and human renal glomerular endothelial cells (HRGEC). For each cell line, at least two culture flasks were maintained for each round of experiment. When the cells reached ∼70% confluency, the supernatant of *P. knowlesi* culture (source of parasite antigens) was mixed with the ECM medium in a 1:9 ratio, and the mixture of media was given to one flask (“primed” group). Another flask worked as the control [the “unexposed” group (unstimulated/naïve)], where the *P. knowlesi* culture supernatant fraction was replaced with unused, batch-matching parasite culture media. Cells were further cultivated for 48 h. Subsequently, the cells were detached with StemPro™ Accutase™ cell dissociation reagent, to be seeded into the RTC-coated eight-well LABTEK chamber slides (each well was seeded with 2 × 10^5^ cells). Cells were cultured with the ECM-“antigen/control” cocktails accordingly. *P. knowlesi* culture suspension (2.5% late stage parasitemia, 0.5% hematocrit) was added into each cell line-seeded well gently 24 h later. The cellular mixtures were incubated for 1 h at *in vitro* cultivation conditions. Non-attached RBCs were gently washed away with media (four times). This was followed by fixation with cold absolute methanol for 10 min. The chambers were removed from the chamber slides, and slides were stained with Giemsa (5%) for 20 min prior to examination using a light microscope under ×1,000 magnification. IRBC–endothelial cell line cytoadherence rate was determined as the number of IRBCs attached on the endothelial cells per 100 fields (equivalent to coverage of ∼8,000 cells). A total of 12 biological replicates were conducted (repeated experiment with different batches of parasites on different batches of endothelial cells primed with supernatant from different batches of parasite cultures). Subsequently, the binding assays on the “primed” cell lines were repeated with IRBC treated with trypsin of different concentrations (10 μg/ml and 1 mg/ml) as elaborated earlier.

### Antibody Blocking Cytoadherence Assay

The binding assay was repeated with slight modification, where antibodies against various reported *P. falciparum* cytoadherence receptors on endothelial cell surface (as listed in Supplementary Table 2) were added. Antibody working concentration of 25 μg/ml was used in all assays. Six biological replicates were conducted for each experiment.

### Characterization of Cell Surface Protein Expression

The cell lines of “unexposed” and “primed” conditions were prepared as described above. Cells were detached and live/dead staining on the cells was performed using LIVE/DEAD™ fixable aqua dead cell stain kit, following the instructions of the manufacturer. Cells were incubated with the antibodies used for antibody blocking assay described above (at a final concentration of 25 μg/ml) suspended in flow cytometry (FACS) buffer (0.5% BSA, 2 mM EDTA in 1× PBS) for 20 min at room temperature. Cellular mixtures were then centrifuged, supernatant was removed and re-suspended to be stained with the secondary antibody goat-anti-rabbit IgG (H + L) cross-adsorbed secondary antibody (PE-conjugated) for 20 min at room temperature. Samples were then analyzed by flow cytometry. For each sample, 3 × 10^4^ events were acquired. At least five biological replicates were done for each experiment.

### Experiments With CD 36-Expressing Chinese Hamster Ovarian Cell Line (CHO-CD36)

The *P. knowlesi*-IRBC binding assay was repeated with CHO cell line expressing human CD36 (CHO-CD36), with CHO-745 cell line as the negative control, as described in earlier cytoadherence studies with malaria parasites *Plasmodium vivax* and *P. falciparum* (Carvalho et al., 2010; Hempel et al., 2017). Briefly, the cells were seeded onto the LABTEK chamber slides (each well was seeded with 1 × 10^5^ cells; coating treatment not required). After overnight incubation, the IRBC cytoadherence assay was conducted as described with the human endothelial cell lines.

### Heparinase Treatment on Human Pulmonary Microvascular Endothelial Cell

The HPMECs were seeded onto LABTEK chamber slides as described earlier. However, RTC was not used for surface coating. Instead, gelatin coating was employed as described elsewhere (Vogt et al., 2003). The seeded cell lines were divided into “unexposed” and “primed” groups. For each group, two sets of cell lines were treated with heparinase I and III blend (working concentration 0.2 IU/ml) for 2 h at *in vitro* cultivation conditions prior to removal of the enzyme from the surface of the cell line. After that, parasite culture suspension and anti-CD36 antibody (working concentration 25 μg/ml) were added accordingly to form these experiment settings: “unexposed, non-haparinase treated,” “unexposed, non-heparinase treated + anti-CD36,” “unexposed, heparinase treated,” “unexposed, heparinase treated + anti-CD36,” “primed, non-heparinase treated,” “primed, non-heparinase treated + anti-CD36,” “primed, heparinase treated,” and “primed, heparinase treated + anti-CD36.” Nine biological replicates were conducted.

### Statistical Analyses

Statistical analyses were performed with GraphPad Prism 9.0. For comparison between two independent groups with nonparametric data distribution, Mann–Whitney test was used. To compare matched/paired nonparametric dataset, Wilcoxon matched pairs signed rank test was used. To compare multiple independent groups with nonparametric distribution, Kruskal–Wallis with Dunn’s multiple comparison test was performed. Comparisons were done with two-sided testing.

## Results

### Rosetting Phenomenon of *Plasmodium knowlesi*-Infected Erythrocytes

**FIGURE 1:**
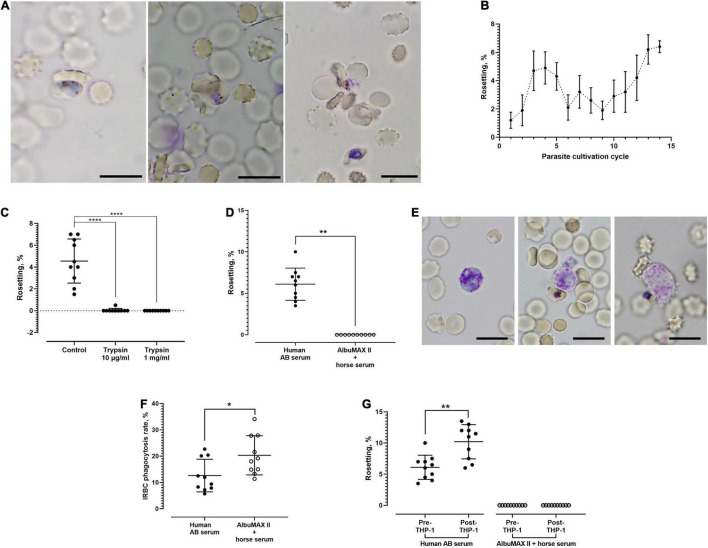
Rosetting phenomenon of *Plasmodium knowlesi* A1-H.1 strain. **(A)** Rosettes formed by *P. knowlesi* A1-H.1 strain, showing small rosettes with involvement of only one (left), three (middle), and five (right) uninfected erythrocytes (URBCs), respectively. Wet mount preparation from 5% Giemsa staining, ×1,000 magnification. Scale bars represent 10 μm. **(B)** Trend of rosetting rates of *P. knowlesi* A1H1 culture across 14 consecutive culture cycles. For each cycle, five replicates were done (mean and SD shown). The starting point (rosetting rate at first cycle post-thawing) was low, probably due to long-term cultivation with AlbuMAX previously, or the rheology of RBC was not well preserved during the cryopreservation and thawing processes. The drop in rosetting rates from during fifth to ninth cycles might be due to different batches of sera used as culture medium enrichment. From the Kruskal–Wallis test, rosetting rates of the 13th cycle were significantly higher than those of the 1st (*p* = 0.0018), 2nd (*p* = 0.0319), and 9th (*p* = 0.0339) cycles. Rosetting rates of the 14th cycle were higher than those of the 1st (*p* = 0.0011), 2nd (*p* = 0.0216), 6th (*p* = 0.0496), and 9th (*p* = 0.0229) cycles. **(C)** Effects of trypsin on rosetting by *P. knowlesi*. The infected erythrocytes (IRBCs) were treated with trypsin of 10 μg/ml and 1 mg/ml prior to mixing with untreated URBCs for rosetting assay. From the Kruskal–Wallis test, significant difference was found between the control and both trypsin treatment groups (*p* < 0.0001 for both groups). **(D)** Rosetting rates of *P. knowlesi* cultures suspended in human AB serum-enriched medium and medium enriched with AlbuMAX II + horse serum (human serum free). The mean of rosetting rates recorded in “human AB serum” group was 6.1% (SD 1.94), whereas no rosette was found in the human serum-free setting (Wilcoxon matched pairs signed rank test *p* = 0.0020). **(E)** A THP-1 cell in resting mode (left), a THP-1 cell initiating engulfment of an IRBC (middle), and a THP-1 in the process of IRBC engulfment (right). Wet mount preparation from 5% Giemsa staining, ×1,000 magnification. Scale bars represent 10 μm. **(F)** IRBC phagocytosis by the THP-1 phagocytes in media with and without human sera. IRBCs suffered higher phagocytosis (20.32 ± 7.439 vs. 12.62 ± 6.21%) in the medium enriched with AlbuMAX II + horse serum, compared with that of human serum-enriched medium (Wilcoxon matched pairs signed rank test *p* = 0.0273). **(G)** Rosetting rates of parasites before and after co-incubation with THP-1 in the media enriched either with human AB serum or AlbuMAX II + horse serum. In the medium enriched with human AB serum, rosetting rates of the parasites increased significantly (from 6.1 ± 1.94 to 10.2 ± 2.741%) after co-incubation with THP-1 (Wilcoxon matched pairs signed rank test *p* = 0.0020). No rosette was found in the human serum-free medium settings before and after THP-1 coincubation. Error bars in all graphs represent mean and SD. *P < 0.05, **P < 0.01, ****P < 0.0001.

Rosettes were not observed when the parasites were cultured in media enriched with AlbuMAX II + horse serum. Rosettes (Figure 1A) started to form when the parasite cultures were maintained in human serum-enriched media. Nevertheless, rosetting were only observed with mature stage parasites (late-stage IRBC with visible malaria pigments). The rosettes were small, consisting of up to six URBC. Throughout the culture follow-up (14 cycles), the rosetting rates fluctuated between 0.5 and 7% (Figure 1B). Rosetting was highly sensitive to trypsin treatment. Even treatment with a low concentration (10 μg/ml) of trypsin completely abrogated rosetting (Figure 1C). No rosettes were found after the IRBCs were treated with 1 mg/ml of trypsin. When we replaced the human serum-enriched media with human serum-free (AlbuMAX II + horse serum-enriched) media, the rosetting capacity of parasite cultures that had stably demonstrated rosetting for consecutive cycles of cultures disappeared (Figure 1D), indicating that the rosetting phenomenon of *P. knowlesi* is human serum dependent. Phagocytosis of IRBC by THP-1 cells (Figure 1E) was less efficient when parasites were grown in human AB serum-enriched media, which facilitated rosette formation (Figure 1F). Co-incubation with THP-1 cells increased the rosetting rates of *P. knowlesi* in human serum-enriched media, whereas rosetting remained absent in the human serum-free settings after co-incubation with THP-1 (Figure 1G).

### Infected Erythrocytes-Endothelial Cytoadherence Assay

**FIGURE 2:**
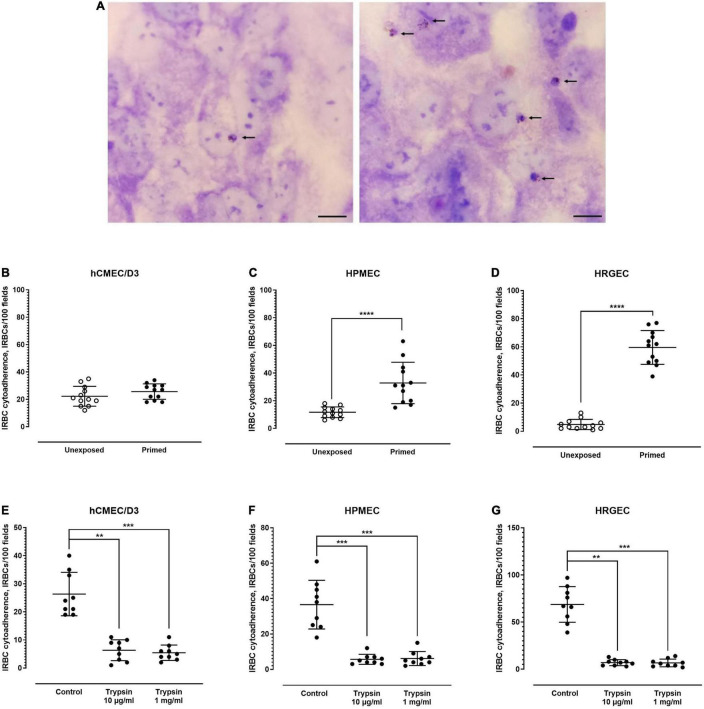
Endothelial cytoadherence properties of *P. knowlesi*-IRBC. **(A)** *Plasmodium knowlesi* IRBC–endothelial cytoadhesion. Snap shots of IRBCs (arrows) adhered to unprimed endothelial cells (control) (left), and endothelial cells primed with culture supernatant of malaria culture (right) in random single field of light microscopy under ×1,000 magnification. All endothelial cells show the cobblestone arrangement under two-dimensional culture as shown in these pictures. This set of pictures were from experiments conducted on human pulmonary microvascular endothelial cells (HPMEC). Scale bars: 10 μm. *P. knowlesi*-IRBC binding to endothelial cell lines derived from different organs, i.e., hCMEC/D3 from the brain **(B)**, HPMEC from the lungs **(C)**, and HRGEC from the kidneys **(D)**. Error bars depict mean and SD. Mann–Whitney test was conducted to compare the binding rate between the unexposed state and post-exposure to *P. knowlesi* antigens in culture supernatant. No significant difference was found for hCMEC/D3 (IRBC binding of 22.25 ± 7.238 and 25.67 ± 5.646 for the unexposed and primed settings, respectively; *p* = 0.2463, *U* = 51.5) **(B)**. Significant increase in IRBC–HPMEC binding was found in the primed group when compared with the unexposed group (IRBC binding of 11.67 ± 3.869 and 23.67 ± 5.789 for the unexposed and primed settings, respectively; *p* < 0.0001, *U* = 4) **(C)**. The primed HRGEC demonstrated higher IRBC binding than its unexposed counterpart (IRBC binding of 4.833 ± 3.639 and 59.58 ± 12.03 for the unexposed and primed settings, respectively; *p* < 0.0001, *U* = 0) **(D)**. **(E–G)** Trypsin sensitivity of IRBC–endothelial binding; error bars represent mean and SD. Kruskal–Wallis with Dunn’s multiple comparison test was conducted. For hCMEC/D3 **(E)**, IRBC binding was significantly lower with the 10-μg/ml (*p* = 0.0017) and 1-mg/ml (*p* = 0.0005) trypsin treatment groups than the untreated control. The IRBC cytoadherence from both trypsin treatment groups were not significantly different from each other (*p* > 0.9999). For HPMEC **(F)**, IRBC cytoadherence was significantly reduced in the trypsin treatment settings when compared with the control (*p* = 0.0008 and 0.0009 for the 10-μg/ml and 1-mg/ml trypsin groups, respectively). No significant difference was found between both trypsin treatment settings (*p* > 0.9999). With HRGEC **(G)**, lower IRBC cytoadherence was recorded with the trypsin treatment groups relative to the control (*p* = 0.0016 and 0.0005 for 10-μg/ml and 1-mg/ml trypsin groups, respectively), and there was no significant difference in the IRBC cytoadherence between the two trypsin treatment settings (*p* > 0.9999). **P < 0.01, ***P < 0.001, ****P < 0.0001.

Under unexposed condition, IRBC adhered minimally to all endothelial cell lines tested (Figures 2A–D). Out of the 100 microscopic fields examined, an average of 22, 12, and 5 IRBCs were found to be adhered to the unexposed brain-derived hCMEC/D3 (Figure 2B, left panel), lung-derived HPMEC (Figure 2C, left panel), and kidney-derived HRGEC (Figure 2D, left panel), respectively. After priming with parasite culture supernatant, hCMEC/D3 did not experience significant change in its IRBC binding (Figure 2B, right panel). On the other hand, the level of IRBC cytoadherence was higher with the primed HPMEC (Figure 2C, right panel) and primed HRGEC (Figure 2D, right panel), compared with their unexposed counterparts. Next, we evaluated the trypsin sensitivity of the *P. knowlesi*-derived ligands involved in endothelial cytoadherence. Similar to its rosetting phenomenon, the *P. knowlesi*-IRBC–endothelial cytoadherence was sensitive to trypsin treatment as low as 10 μg/ml, and there was no significant difference in IRBC binding between the 10-μg/ml and 1-mg/ml trypsin treatments for all the endothelial cell lines tested (Figures 2E–G).

### Antibody Blocking Cytoadherence Assay

**FIGURE 3:**
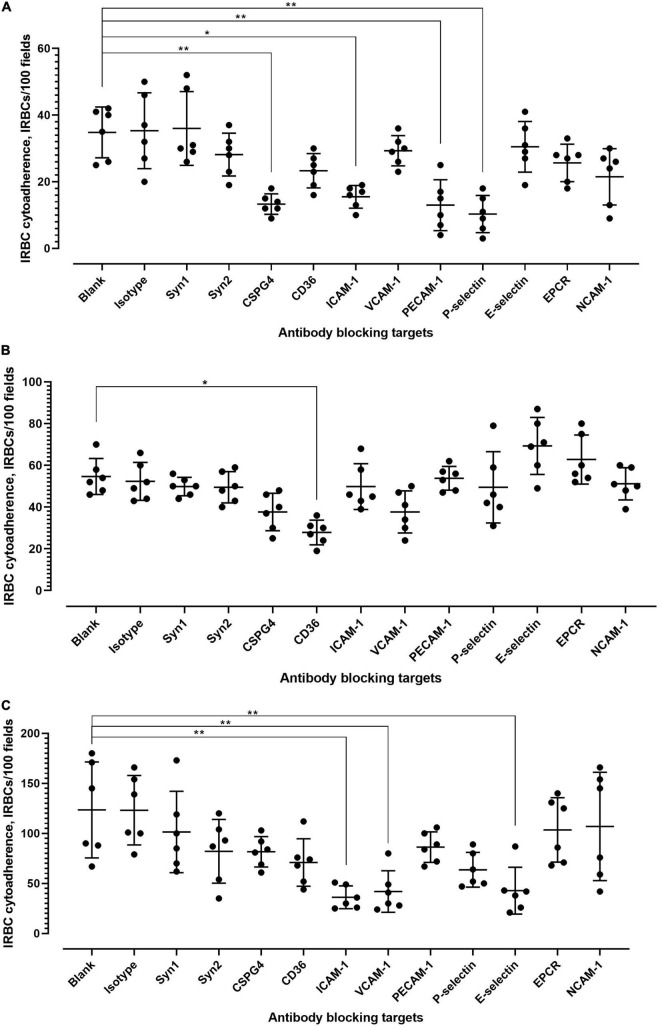
Antibody blocking of IRBC–endothelial cytoadherence. **(A)** Effect of antibody blocking on *P. knowlesi* IRBC cytoadherence to primed hCMEC/D3. Error bars represent mean and SD of data points collected from six biological replicates. Kruskal–Wallis with Dunn’s multiple comparison test was conducted to evaluate the binding blocking effect of each antibody relative to the antibody-free blank setting. Significant inhibition was found for antibody blocking of CSPG4 (chondroitin sulfate) (adjusted *p* = 0.0048), ICAM1 (adjusted *p* = 0.0197), PECAM1 (adjusted *p* = 0.0063), and P-selectin (adjusted *p* = 0.0015). **(B)** Effect of antibody blocking on *P. knowlesi* IRBC cytoadherence to primed HPMEC. Error bars represent mean and SD of data points collected from six biological replicates. Kruskal–Wallis with Dunn’s multiple comparison test was conducted to evaluate the binding blocking effect of each antibody relative to the antibody-free blank setting. Significant inhibition was found for antibody blocking of CD36 (adjusted *p* = 0.0110). **(C)** Effect of antibody blocking on *P. knowlesi* IRBC cytoadherence to primed HPMEC. Error bars represent mean and SD of data points collected from six biological replicates. Kruskal–Wallis with Dunn’s multiple comparison test was conducted to evaluate the binding blocking effect of each antibody relative to the antibody-free blank setting. Significant inhibition was found for antibody blocking of ICAM1 (adjusted *p* = 0.0023), VCAM1 (adjusted *p* = 0.0071), and E-selectin (adjusted *p* = 0.0094). *P < 0.05, **P < 0.01.

Various receptors are involved in the cytoadherence of *P. falciparum*-IRBC to endothelial cells (Supplementary Table 2). We conducted antibody blocking assay to determine whether these receptors were also involved in *P. knowlesi*-IRBC cytoadherence. Given that the IRBC cytoadherence on “unprimed” endothelial cells was low, experiments were conducted with primed endothelial cell lines. For hCMEC/D3 (Figure 3A; detailed statistical analyses are available in Supplementary Table 3), significant inhibition of IRBC–endothelial cytoadherence was found with the antibody blocking of chondroitin sulfate (CSPG4), ICAM-1, PECAM-1, and P-selectin. With HPMEC (Figure 3B; detailed statistical analyses are available in Supplementary Table 4), antibody blocking of CD36 significantly reduced the IRBC–endothelial binding. For HRGEC (Figure 3C; detailed statistical analyses are available in Supplementary Table 5), significant IRBC–endothelial binding inhibition was found with antibody blocking of ICAM-1, VCAM-1, and E-selectin. The isotype controls did not exert significant changes to the IRBC–endothelial binding.

### Receptor Expression Profile on the Surface of Endothelial Cells

Using flow cytometry, we investigated whether *P. knowlesi* culture supernatant exposure modified the surface expression profile of the cytoadherence receptors by the endothelial cell population. For the 11 protein candidates under study, the percentage of hCMEC/D3 cells with surface expression of these proteins was not significantly modified by *P. knowlesi* culture supernatant exposure (Supplementary Figure 1; detailed statistical analyses in Supplementary Table 6). With HPMEC, the percentage of cells expressing syndecan-2 and VCAM-1 were significantly reduced (Supplementary Figure 2; detailed statistical analyses in Supplementary Table 7). Similar results to that of hCMEC/D3 were made for HRGEC (Supplementary Figure 3; detailed statistical analyses in Supplementary Table 8).

**FIGURE 4:**
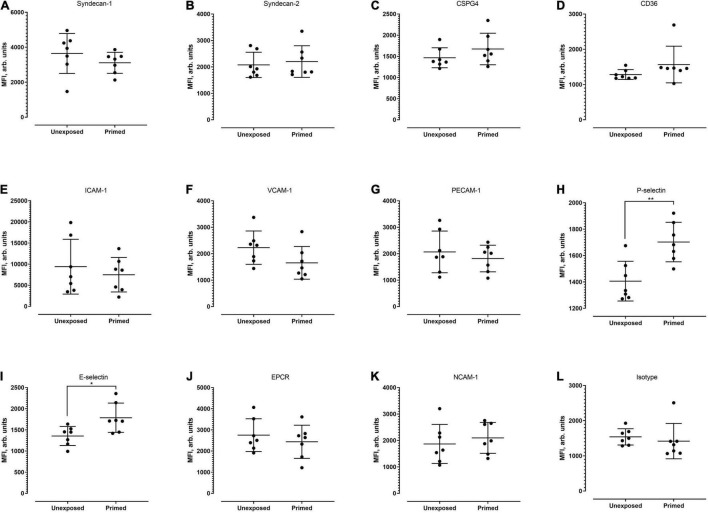
Median fluorescence intensity (MFI) profiles of each of the receptors studied **(A–L)** on hCMEC/D3. Seven biological replicates were conducted. Based on Mann–Whitney test, signal intensity was significantly higher for P-selectin (*p* = 0.0070) and E-selectin (*p* = 0.0286). *P < 0.05, **P < 0.01.

**FIGURE 5:**
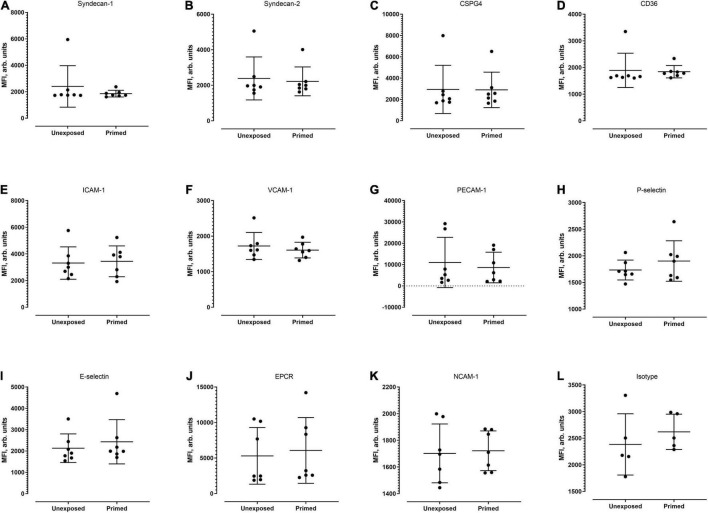
Median fluorescence intensity profiles of each of the receptors studied **(A–L)** on HPMECs. Seven biological replicates were conducted (except for isotype group, which was of five replicates). Based on Mann–Whitney test, there was no significant difference in MFI profiles of all candidates between the unexposed and exposed settings.

**FIGURE 6:**
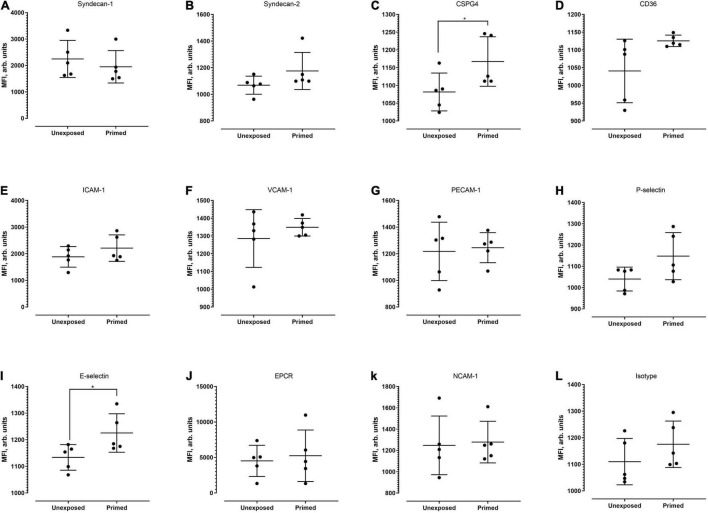
Median fluorescence intensity profiles of each of the receptors studied **(A–L)** on HRGEC. Five biological replicates were conducted. Based on Mann–Whitney test, signal intensity of CSPG-4 and E-selectin was significantly higher in the primed group (*p* = 0.0476 and 0.0317, respectively). *P < 0.05.

Next, we examined whether the level of cell surface protein expression, as measured by the median fluorescence intensity (MFI) was altered following exposure to *P. knowlesi* culture supernatant. For hCMEC/D3 cells, priming significantly increased the MFI of P-selectin and E-selectin (Figure 4; detailed statistical analyses in Supplementary Table 9). For HPMEC cells, MFI profiles of the different proteins studied were not significantly altered following *P. knowlesi* culture supernatant exposure (Figure 5; detailed statistical analyses in Supplementary Table 10). For HRGEC cells, priming significantly increased the MFI of CSPG4 and E-selectin (Figure 6; detailed statistical analyses in Supplementary Table 11). The findings from antibody blocking assay and FACS are summarized in Table 1.

**TABLE 1:** Summary of receptors involved in *Plasmodium knowlesi* infected erythrocytes (IRBC) cytoadherence to different endothelial cell lines after priming with *P. knowlesi* culture supernatant.

1.1.1	Endothelial cell lines		
	hCMEC/D3	HPMEC	HRGEC
Receptor involved in IRBC cytoadherence^1^	CSPG4 ICAM-1 VCAM-1 PECAM-1 P-selectin	CD36	ICAM-1 VCAM-1 E-selectin
Receptors whose prevalence of surface expression by endothelial cell population significantly altered post-priming^2^	N/A	Syndecan-2 (reduced), VCAM-1 (reduced)	N/A
Receptor whose surface expression level on individual cells increased post-priming^3^	P-selectin E-selectin	N/A	CSPG-4 E-selectin

*N/A, not available; HPMEC, human pulmonary microvascular endothelial cells.*

*^1^Determined using antibody blocking assays.*

*^2^Determined by flow cytometry.*

*^3^Determined from median fluorescence intensity (MFI) via flow cytometry.*

**FIGURE 7:**
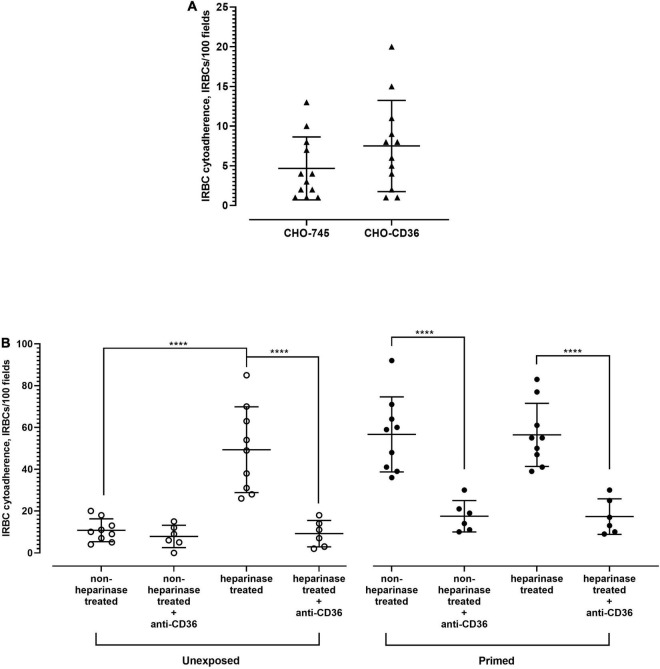
Role of CD36 in *P. knowlesi* IRBC binding to HPMEC. **(A)** Chinese hamster ovarian (CHO) cell line surface-expressing human CD36 (CHO-CD36) and control (CHO-745) were incubated with *P. knowlesi* culture suspension. From Mann–Whitney test, no significant difference in IRBC cytoadhered to both cell lines (*p* = 0.2153). **(B)** HPMEC cell lines (unexposed and primed with *P. knowlesi* culture supernatant) were recruited for IRBC binding assay. Each group of cell line was divided into heparinase- and non-heparinase-treated groups, each of which was further divided into two conditions, where one was incubated with anti-CD36 antibody and the other half served as antibody-free setting. One-way ANOVA with Tukey’s test was done for statistical comparison. Unexposed cell line, which had low IRBC binding, experienced significantly higher IRBC binding with heparinase-treatment setting (*p* < 0.0001), which was significantly hampered by anti-CD36 antibody (*p* < 0.0001). The unexposed cell lines treated with heparinase experienced IRBC–HPMEC cytoadherence similar to that of HPMEC primed with parasite culture supernatant (*p* = 0.9335). For the primed cell line, no significant difference in IRBC binding was found between the heparinase and non-heparinase treatment settings (*p* > 0.9999). Anti-CD36 antibody significantly reduced IRBC binding to both heparinase-treated and untreated cell lines (*p* < 0.0001 for both settings). From the comparison with “primed, non-heparinase treated + anti-CD36,” no significant difference was found for the “unexposed, heparinase treated + anti-CD36” (*p* = 0.9547) and “primed, heparinase treated + anti-CD36” (*p* > 0.9999). ****P < 0.0001.

Among the receptor candidates tested, CD36 was the only candidate whose antibody blockade significantly inhibited IRBC–HPMEC binding. However, the surface CD36 expression profile of HPMEC cells was not altered by the priming step, and the *P. knowlesi*-IRBC did not bind more to the CHO-CD36, compared with the negative control CHO-745 cell line (Figure 7A). It was also revealed that the “priming” step reduced the number of HPMEC expressing syndecan-2. Previously, glycocalyx was shown to hamper the binding of *P. falciparum*-IRBC to CD36 (Hempel et al., 2017). Syndecan-2 is one of the key components of endothelial glycocalyx (Van Teeffelen et al., 2007; Zeng, 2017; Masola et al., 2021). Hence, we investigated if enzymatic removal of syndecan-2 on HPMEC would influence the IRBC–HPMEC binding. As mentioned earlier, HPMEC cells that were not exposed to culture supernatant of *P. knowlesi* demonstrated low IRBC binding. However, treatment of these unexposed cell lines with heparinase prior to incubation with IRBC significantly increased the IRBC–HPMEC cytoadherence to a similar extent as the HPMEC primed with parasite culture supernatant (Figure 7B). The heparinase-facilitated IRBC–HPMEC binding was significantly hampered by anti-CD36 antibody. For HPMEC cells primed with parasite culture supernatant, heparinase treatment did not significantly increase the IRBC cytoadherence, compared with the non-heparinase-treated HPMECs that were primed with parasite culture supernatant. Among the experiment settings involving anti-CD36 antibody, when comparing against the setting of “primed, non-heparinase treated + anti-CD36,” no significant difference was found in the IRBC–HPMEC cytoadherence for both “unexposed, heparinase treated + anti-CD36” and “primed, heparinase treated + anti-CD36,” suggesting that the enhanced IRBC binding from heparinase treatment was attributed to CD36.

## Discussion

Here, we have characterized the cytoadherence properties of *P. knowlesi* A1-H.1 reference strain. First, rosette formation happened when the parasites developed to malaria pigment-containing late stages, similar to that of *P. falciparum* and *P. vivax* (Udomsangpetch et al., 1989; Lee et al., 2014). Nevertheless, the frequency of rosette formation by *P. knowlesi* (at least for the A1-H.1 strain) was relatively low. This may be due to several reasons. First, the parasites were adapted to human serum-free cultivation for a long period of time prior to this study. Previously, long-term human serum-free cultivation of *P. falciparum* has been reported to reduce the knob formation on the surface of IRBC (Langreth et al., 1979), which are responsible for a significant part of the cytoadherence characteristics by *P. falciparum*-IRBC (Horrocks et al., 2005). Although knob formation does not occur on *P. knowlesi*-IRBC (Russell and Cooke, 2017), the availability of human serum may still influence the cytoadherence ligand expression profile of *P. knowlesi*-IRBC. Of note, the importance of human serum to *P. falciparum* rosetting has been demonstrated (Luginbuhl et al., 2007). In fact, we did not observe any rosette in the human serum-free *P. knowlesi* cultures until after the parasites were adapted to human serum-containing cultivation conditions. Furthermore, the rosettes formed by the parasites in human serum-containing media dissociated when the parasites were suspended in human serum-free media, highlighting the importance of human serum to *P. knowlesi* rosetting. We also attempted rosetting assay with FBS-enriched RPMI-1640 (culture medium for THP-1 and CHO cells), and it did not support rosette formation. The fluctuation in rosetting rates across the erythrocytic cycles happened concurrently with the usage of human sera from different sources as culture medium enrichment. We do not know the human serum components that influence *P. knowlesi* rosetting and whether they are the same as those reported for *P. falciparum* and *P. vivax* (Luginbuhl et al., 2007; Lee et al., 2020). It is possible that a factor in human serum with varied levels across different sera samples, may influence the rosetting rates of *P. knowlesi*-IRBC. We believe that the longer period of continuous culture for this parasite with the right human sera may further increase its rosetting capacity. In fact, higher rosetting rates were recorded in the phagocytosis assay, suggesting that the parasites possess capacity of forming more rosettes. Functionally, rosetting of *P. knowlesi*-IRBC with URBC hampered phagocytosis of IRBC, which agreed well with earlier findings with *P. falciparum* and *P. vivax* parasites (Albrecht et al., 2020; Lee et al., 2020). Rosetting reduced the phagocytosis of *P. knowlesi*-IRBC by ∼38%. Notably, we conducted all experiments only with RBC of group “O.” Hence, the role of ABO blood groups in *P. knowlesi* rosetting remains to be investigated.

The *P. knowlesi*-IRBC had low affinity to cerebral microvascular endothelial cells. Priming with *P. knowlesi* culture supernatant did not significantly increase the binding of IRBC to these endothelial cells. This corresponds with the clinical pathological profiles seen in knowlesi malaria, where coma due primarily to cerebral malaria have not been reported (Daneshvar et al., 2009; William et al., 2011). On the other hand, primed lung and kidney endothelial cells bound more IRBC. Nevertheless, further studies with more clinical isolates are required to validate if there is a binding tropism by *P. knowlesi*. In this study, we have shown that endothelial cells (at least for HPMEC and HRGEC cells) can be “primed” (for enhanced IRBC–endothelial cytoadherence) independently of direct physical contact between the endothelial cells and *P. knowlesi*-IRBC. With *P. falciparum*, direct contact between IRBC and endothelial cells (Viebig et al., 2005), as well as malaria pigments (hemozoin) have been shown to activate endothelial cells (Basilico et al., 2017). Indeed, *P. knowlesi* culture supernatant that we used for endothelial cell priming contained the parasite-derived antigens (including the hemozoin) and extracellular hemoglobin following hemolysis. Interestingly, evidence suggestive of hemolysis-mediated endothelial activation in knowlesi malaria patients has been reported previously (Barber et al., 2018a). In knowlesi malaria, endothelial activation may occur ahead of IRBC–endothelial cytoadherence. Nevertheless, more work is needed to decipher the nature of the priming agents and the sequence of the pathological events in *P. knowlesi* infection.

Significant IRBC–endothelial binding inhibition was achieved with antibodies specific to different receptors on different types of endothelial cells (Table 1). It is worthwhile to point out that *P. knowlesi* clinical isolates were shown to have low binding affinity to the recombinant human Fc chimeric CD36 coated on a Petri dish (Fatih et al., 2012). In our study, the priming step altered neither the percentage of HPMEC cells expressing CD36 nor its intensity, but reduced the population of HPMECs that expressed syndecan-2, a crucial component of endothelial glycocalyx (Martinez-Seara Monne et al., 2013). When we disrupted the HPMEC glycocalyx integrity by removing syndecans with heparinase, even the non-exposed HPMEC experienced increased IRBC binding like the primed HPMEC, and the binding of IRBC to these “non-exposed but heparinase-treated” HPMEC was inhibited by the anti-CD36 antibody. The “priming step” may disrupt the glycocalyx integrity of HPMEC, which allows better interactions between CD36 and the *P. knowlesi*-derived cytoadherence ligands. When we re-evaluated the importance of CD36 in *P. knowlesi*-IRBC cytoadherence using the human CD36-expressing CHO cell line, the *P. knowlesi*-IRBC did not show higher cytoadherence to the CHO-CD36 cell line than the control cell line, which tallied with the earlier findings (Fatih et al., 2012). Notably, human CD36 is a highly glycosylated protein (Hoosdally et al., 2009; Meyre et al., 2019). The posttranslational modification of a protein by human cells is different from those of vectors that produce recombinant proteins (Jarvis et al., 1998; Du et al., 2019). Although the posttranslation machinery of the mammalian CHO cells is much closer to that of human cells, CHO cell lacks certain glycosylation-involving enzymes expressed by humans cells, which may result in different glycosylation outcomes (Goh and Ng, 2018). This may contribute to the contradicting results with different experimental approaches. Given that the sugar moiety of human CD36 may influence *P. knowlesi*-IRBC-CD36 binding, the binding domains and requirements of CD36 involved in cytoadherence to *P. knowlesi*-IRBC may be different from those of *P. falciparum* and *P. vivax* (Carvalho et al., 2010; Janes et al., 2011).

Another intriguing finding from this study was the different outcomes of priming with *P. knowlesi* culture supernatant for different human endothelial cell lines. Indeed, the distinct expression profiles of endothelial cells derived from different types of vasculature and organs have been demonstrated previously (Chi et al., 2003). More studies are required to investigate the mechanisms behind the differential expression responses to *P. knowlesi* antigens by different endothelial cells, which will assist further understanding on organ-specific pathogenesis in knowlesi malaria. As for the parasite-derived ligands involved in these cytoadherence events, *P. knowlesi* has been shown to express variant antigens on its IRBC (SICAvar) (Howard et al., 1983). Nevertheless, the roles of SICAvar remain to be validated. The *P. knowlesi*-derived cytoadherence ligands may consist of one type of variant antigen with different binding domains for different receptors, or several parasite proteins with distinct binding receptors. These yet to be identified ligands are highly sensitive to trypsin. Taken together, we have characterized the basic cytoadherence properties of *P. knowlesi* A1-H.1 reference strain, layering a foundation for further investigations on the interactive dynamics between the *P. knowlesi*-IRBC and the host-derived cells in vasculature.

## Data Availability Statement

The original contributions presented in the study are included in the article/Supplementary Material, further inquiries can be directed to the corresponding authors.

## Author Contributions

W-CL, Y-LL, and LR conceived the project and compiled, analyzed, and interpreted the data. W-CL, Y-LL, and SS conceptualized and planned the experiments. W-CL and SS prepared and managed the fieldwork. W-CL, SS, and SN prepared, managed, and performed the experiments. Y-LL and LR managed the ethical clearance, processing of the samples and logistic matters. W-CL, SS, LR, and Y-LL prepared the manuscript. All authors read and approved the final manuscript.

## Conflict of Interest

The authors declare that the research was conducted in the absence of any commercial or financial relationships that could be construed as a potential conflict of interest.

## Publisher’s Note

All claims expressed in this article are solely those of the authors and do not necessarily represent those of their affiliated organizations, or those of the publisher, the editors and the reviewers. Any product that may be evaluated in this article, or claim that may be made by its manufacturer, is not guaranteed or endorsed by the publisher.
