# Supplementary material for: Cytoadherence Properties of Plasmodium knowlesi-Infected Erythrocytes
Source: Front Microbiol. 2022 Jan 5;12:804417. doi: 10.3389/fmicb.2021.804417 (PMC8767020; doi:10.3389/fmicb.2021.804417)
Supplement: Supplementary file 1 [file Data_Sheet_1.docx]

**Supplementary Material**

**Supplementary table 1. Information on tools, reagents and biological materials used in this study.**

| **Reagent/Resource** | **Reference or source** | **Identifier/ Catalog Number / Notes** |
| --- | --- | --- |
| **Biological materials** |  |  |
| A1-H.1 (*Plasmodium knowlesi*) | Laboratory-adapted parasite strain; maintained in University of Malaya and A*STAR ID labs.  Moon *et al*.2013.doi: 10.1073/pnas.1216457110 | Used for all experiments. |
| CHO-CD36 cell line | Carvalho *et al*. 2010.doi:10.1086/654815; maintained in University of Malaya and A*STAR ID labs. | Used for binding assay. |
| CHO-745 cell line | Carvalho *et al*. 2010.doi:10.1086/654815; maintained in University of Malaya and A*STAR ID labs. | Used for binding assay. |
| hCMEC/D3 (human cerebral microvascular endothelial cells) | A kind gift of Pierre Olivier Couraud, Institut Cochin, Paris, France; maintained in A*STAR ID labs. | Used for all cytoadherence assay. |
| HPMEC (human pulmonary microvascular endothelial cells) | ScienCell™ Research Laboratories;  maintained in A*STAR ID labs. | Cat# 3000; used for all cytoadherence assay. |
| HRGEC (human renal glomerular endothelial cells) | ScienCell™ Research Laboratories;  maintained in A*STAR ID labs. | Cat# 4000; used for all cytoadherence assay. |
| THP-1 cell line | ATCC; maintained in University of Malaya and A*STAR ID labs. | Cat# TIB-202^™^; human monocytic cell line; used for phagocytosis assay. |
| **Antibodies** |  |  |
| Rabbit anti-humanCspg4 polyclonal IgG | Sino Biological | Cat#100947-T32; targeted receptor: chondroitin sulfate |
| Rabbit anti-human Syndecan I polyclonal IgG | Sino Biological | Cat# 11429-RP02; targeted receptor: heparan sulfate proteoglycan |
| Rabbit anti-human Syndecan II polyclonal IgG | Sino Biological | Cat#310259-T10; targeted receptor: heparan sulfate proteoglycan |
| Rabbit anti-human CD36 polyclonal IgG | Sino Biological | Cat#10752-RP02; targeted receptor: platelet glycoprotein 4 (CD36) |
| Rabbit anti-human ICAM1 polyclonal IgG | Sino Biological | Cat # 10346-T26; targeted receptor: intercellular adhesion molecule 1 (ICAM-1/CD54) |
| Rabbit anti-human VCAM1 polyclonal IgG | Sino Biological | Cat#10113-T56; targeted receptor: vascular cell adhesion protein molecule 1 (VCAM-1/ CD106) |
| Rabbit anti-human CD31 polyclonal IgG | Sino Biological | Cat#10148-T60; targeted receptor: platelet endothelial cell adhesion molecule 1 (PECAM-1/CD31) |
| Rabbit anti-human CD62P polyclonal IgG | Sino Biological | Cat#13025-RP01; targeted receptor: P-selectin |
| Rabbit anti-human CD62E polyclonal IgG | Sino Biological | Cat#10335-T16; targeted receptor: E-selectin |
| Rabbit anti-human EPCR polyclonal IgG | Sino Biological | Cat #13320-RP02; targeted receptor: Endothelial protein C receptor (EPCR/CD201) |
| Rabbit anti-human NCAM1 polyclonal IgG | Sino Biological | Cat#10673-T26; targeted receptor: Neural cell adhesion molecule 1 (NCAM-1) |
| Normal rabbit isotype control IgG | Sino Biological | Cat # CR1; isotype antibody |
| Goat-anti-rabbit IgG (H+L) cross-adsorbed secondary antibody, PE | Invitrogen | Cat#P-2771MP |
| **Chemicals, Enzymes and other reagents** |  |  |
| 1X phosphate buffer saline (PBS) | Gibco™ | Cat# 20012-027 |
| Acetic acid (glacial) | Merck | Cat#1.00063.2500 |
| AlbuMAX II™ | Gibco™ ThermoFisher Scientific | Cat#11021-045 |
| BD Vacutainer™ with lithium heparin | ThermoFisher Scientific | Cat# 02-657-28 |
| Bovine Serum Albumin (BSA) | Sigma-Aldrich® | Cat#A2153 |
| D-glucose | Sigma-Aldrich® | Cat#47829 |
| Dimethyl sulfoxide (DMSO) | Sigma-Aldrich® | Cat#D2650 |
| Endothelial cell medium (ECM) set | ScienCell™ Research Laboratories | Cat#1001 |
| Ethylenediaminetetraacetic acid disodium salt dihydrate (EDTA-2NA) | Sigma-Aldrich® | Cat#E5134 |
| Fetal Bovine Serum (FBS) | Gibco™ | Cat# 10500 |
| Gelatin solution | Sigma-Aldrich® | Cat#G1393 |
| Giemsa | Merck | Cat# HX60416604 |
| Heparinase I and III blend | Sigma-Aldrich® | Cat# H3917 |
| Hypoxanthine | Calbiochem | Cat#4010CBC |
| Horse serum | Gibco™ | Cat# 16050122 |
| LIVE/DEAD™ fixable aqua dead cell stain kit | Invitrogen™ | Cat# L34957 |
| Methanol | Merck | Cat#1.06009.2500 |
| MycoAlert™ PLUS Mycoplasma detection kit | Lonza | Cat # LT07-705 |
| Rat tail collagen | ThermoFisher Scientific | Cat#A1048301 |
| RPMI 1640 medium | HyClone™ | Cat# SH30255.01 |
| StemPro™ Accutase™ cell dissociation reagent | ThermoFisher Scientific | Cat# A1110501 |
| Trypan blue | Sigma-Aldrich® | Cat#T6146 |
| Trypsin solution | Gibco™ | Cat # 25300054 |
| **Software** |  |  |
| FlowJo | BD Life Sciences |  |
| GraphPad Prism version 9.0 | GraphPad |  |
| **Others** |  |  |
| 70µm nylon mesh cell strainer | Falcon® | Cat#352350 |
| Cellulose acetate filter pore size 0.45 µm | Sartorius Minisart®, Sigma-Aldrich | Cat# 16555-K |
| Centrifuge | Sorvall® | Legend® RT Plus |
| Dual CCD digital camera for microscope | Olympus® | Model DP21 |
| Falcon® Cell Culture Flask T25, filter cap | VWR™ | Cat#29185298 |
| Flow cytometer | BD Life Sciences | Model LSR Fortessa™ |
| Glass coverslip 22x32mm | Mariendfeld | Cat# 0101112 |
| Glass slide | Sail brand | Cat# 7105 |
| LABTEK chamber slides | ThermoFisher Scientific | Cat# 177445 |
| LD columns | Miltenyi Biotec | Cat # 130-042-901 |
| Light microscope | Olympus® | Model BX43 |
| Micronic tubes, 1.40 ml | Micronic | Cat# MP32022 |
| QuadroMACS™ separator | Miltenyi Biotec | Cat # 130-090-976 |

**Supplementary table 2. List of antibodies against the established cytoadherence receptors found for *P. falciparum*.**

| **No.** | **Antibodies** | **Targeted receptor** | **References/ Notes** |
| --- | --- | --- | --- |
| 1 | Rabbit anti-humanCspg4 polyclonal IgG (Sino Biological Cat#100947-T32) | Chondroitin sulfate | (Fried and Duffy, 1996;Khattab et al., 2001) |
| 2 | Rabbit anti-human Syndecan I polyclonal IgG (SinoBiological Cat# 11429-RP02) | Heparan sulfate proteoglycan | (Barragan et al., 2000;Vogt et al., 2003) |
| 3 | Rabbit anti-human Syndecan II polyclonal IgG (SinoBiological Cat#310259-T10) | Heparan sulfate proteoglycan | (Barragan et al., 2000;Vogt et al., 2003) |
| 4 | Rabbit anti-human CD36 polyclonal IgG (SinoBiological Cat#10752-RP02) | Platelet glycoprotein 4 (CD36) | (Turner et al., 1994;Baruch et al., 1997;Yipp et al., 2000) |
| 5 | Rabbit anti-human ICAM1 polyclonal IgG (SinoBiological Cat # 10346-T26) | Intercellular adhesion molecule 1 (ICAM1/ CD54) | (Turner et al., 1994;Udomsangpetch et al., 1997;Smith et al., 2000;Yipp et al., 2000;Armah et al., 2005;Gullingsrud et al., 2013) |
| 6 | Rabbit anti-human VCAM1 polyclonal IgG (SinoBiological Cat#10113-T56) | Vascular cell adhesion protein molecule 1 (VCAM1/CD106) | (Udomsangpetch et al., 1997;Yipp et al., 2000;Armah et al., 2005) |
| 7 | Rabbit anti-human CD31 polyclonal IgG (SinoBiological Cat#10148-T60) | Platelet endothelial cell adhesion molecule 1 (PECAM1/ CD31) | (Treutiger et al., 1997;Berger et al., 2013) |
| 8 | Rabbit anti-human CD62P polyclonal IgG (SinoBiological Cat#13025-RP01) | P-selectin | (Udomsangpetch et al., 1997;Smith et al., 2000;Yipp et al., 2000) |
| 9 | Rabbit anti-human CD62E polyclonal IgG (SinoBiological Cat#10335-T16) | E-selectin | (Turner et al., 1994;Armah et al., 2005) |
| 10 | Rabbit anti-human EPCR polyclonal IgG (SinoBiological Cat #13320-RP02) | Endothelial protein C receptor (EPCR/CD201) | (Bernabeu et al., 2016;Kessler et al., 2017;Shabani et al., 2017) |
| 11 | Rabbit anti-human NCAM1 polyclonal IgG (SinoBiological Cat#10673-T26) | Neural cell adhesion molecule 1 (NCAM1) | (Pouvelle et al., 2007) |
| 12 | Normal rabbit isotype control IgG (Sino Biological Cat # CR1) | Isotype antibody | control |

**Supplementary table 3. Antibody blocking assay on hCMEC/D3 with statistical analyses performed.**

| **Antibody blocking targets** | **Mean (IRBC/ 100 fields)** | **S.D.** | **Kruskal-Wallis with Dunn’s multiple test’s adjusted P value relative to “Blank”** |
| --- | --- | --- | --- |
| Blank | 34.83 | 7.627 | N/A |
| Isotype Ab | 35.33 | 11.38 | > 0.9999 |
| Syndecan 1 | 36.00 | 11.05 | > 0.9999 |
| Syndecan 2 | 28.17 | 6.432 | > 0.9999 |
| CSPG4 | 13.33 | 3.077 | 0.0048 |
| CD36 | 23.33 | 5.164 | 0.8917 |
| ICAM1 | 15.50 | 3.391 | 0.0197 |
| VCAM1 | 29.33 | 4.546 | > 0.9999 |
| PECAM1 | 13.00 | 7.616 | 0.0063 |
| P-selectin | 10.33 | 5.574 | 0.0015 |
| E-selectin | 30.50 | 7.583 | > 0.9999 |
| EPCR | 25.67 | 5.610 | > 0.9999 |
| NCAM1 | 21.50 | 8.456 | 0.4526 |

**Supplementary table 4. Antibody blocking assay on HPMEC with statistical analyses performed.**

| **Antibody blocking targets** | **Mean (IRBC/ 100 fields)** | **S.D.** | **Kruskal-Wallis with Dunn’s multiple test’s adjusted P value relative to “Blank”** |
| --- | --- | --- | --- |
| Blank | 54.67 | 8.641 | N/A |
| Isotype Ab | 52.33 | 9.092 | > 0.9999 |
| Syndecan 1 | 49.83 | 4.401 | > 0.9999 |
| Syndecan 2 | 49.50 | 7.503 | > 0.9999 |
| CSPG4 | 37.67 | 8.959 | 0.1788 |
| CD36 | 27.83 | 5.913 | 0.0110 |
| ICAM1 | 49.83 | 10.94 | > 0.9999 |
| VCAM1 | 37.67 | 10.09 | 0.2441 |
| PECAM1 | 53.83 | 5.707 | > 0.9999 |
| P-selectin | 49.50 | 17.10 | > 0.9999 |
| E-selectin | 69.33 | 13.63 | > 0.9999 |
| EPCR | 62.83 | 11.77 | > 0.9999 |
| NCAM1 | 51.17 | 7.731 | > 0.9999 |

**Supplementary table 5. Antibody blocking assay on HRGEC with statistical analyses performed.**

| **Antibody blocking targets** | **Mean (IRBC/ 100 fields)** | **S.D.** | **Kruskal-Wallis with Dunn’s multiple test’s adjusted P value relative to “Blank”** |
| --- | --- | --- | --- |
| Blank | 123.50 | 47.93 | N/A |
| Isotype Ab | 123.17 | 34.68 | > 0.9999 |
| Syndecan 1 | 101.50 | 40.61 | > 0.9999 |
| Syndecan 2 | 82.17 | 31.83 | > 0.9999 |
| CSPG4 | 81.67 | 15.20 | > 0.9999 |
| CD36 | 71.00 | 23.72 | 0.7019 |
| ICAM1 | 36.17 | 11.41 | 0.0023 |
| VCAM1 | 42.00 | 20.77 | 0.0071 |
| PECAM1 | 86.33 | 15.27 | > 0.9999 |
| P-selectin | 63.67 | 17.37 | 0.2944 |
| E-selectin | 42.83 | 23.39 | 0.0094 |
| EPCR | 103.50 | 32.17 | > 0.9999 |
| NCAM1 | 107.00 | 54.08 | > 0.9999 |

**Supplementary table 6. Comparison of hCMEC/D3 cell populations with surface expression of the receptor candidates of interest between unexposed condition and condition with exposure to *P. knowlesi* culture supernatant.**

| **Receptor candidates** | **Unexposed (%)** | | **A1-H.1 (%)** | | **Mann-Whitney P** |
| --- | --- | --- | --- | --- | --- |
|  | **Mean** | **S.D** | **Mean** | **S.D** |  |
| Syndecan 1 | 69.9700 | 14.1600 | 71.1000 | 10.5300 | 0.9015 |
| Syndecan 2 | 0.7371 | 0.6684 | 0.8200 | 0.8584 | 0.5344 |
| CSPG4 | 0.4800 | 0.2573 | 0.4100 | 0.3281 | 0.4330 |
| CD36 | 0.8171 | 0.1919 | 0.5143 | 0.3253 | 0.1049 |
| ICAM1 | 75.4400 | 5.8320 | 75.6400 | 9.8840 | 0.5350 |
| VCAM1 | 65.3600 | 11.3700 | 56.6900 | 16.9700 | 0.3829 |
| PECAM1 | 60.1600 | 20.9000 | 58.3100 | 22.2600 | 0.8048 |
| P-selectin | 0.2584 | 0.2032 | 0.2183 | 0.1940 | 0.2990 |
| E-selectin | 1.6600 | 1.3570 | 0.6400 | 0.4012 | 0.0728 |
| EPCR | 72.6900 | 8.2110 | 71.1000 | 13.3400 | >0.9999 |
| NCAM1 | 0.3043 | 0.1612 | 0.2457 | 0.1449 | 0.8310 |
| Isotype | 0.4443 | 0.3167 | 0.4157 | 0.4031 | 0.7104 |

**Supplementary table 7. Comparison of HPMEC cell populations with surface expression of the receptor candidates of interest between unexposed condition and condition with exposure to *P. knowlesi* culture supernatant.**

| **Receptor candidates** | **Unexposed (%)** | | **A1-H.1 (%)** | | **Mann-Whitney P** |
| --- | --- | --- | --- | --- | --- |
|  | **Mean** | **S.D** | **Mean** | **S.D** |  |
| Syndecan 1 | 20.9400 | 13.1000 | 17.2600 | 9.0950 | 0.5335 |
| Syndecan 2 | 2.0130 | 1.7890 | 1.0030 | 0.9832 | 0.0466 |
| CSPG4 | 0.6771 | 0.47250 | 8.9260 | 22.8700 | 0.1783 |
| CD36 | 1.2490 | 0.9830 | 0.7000 | 0.3434 | 0.1200 |
| ICAM1 | 43.9000 | 5.1630 | 38.8600 | 7.6320 | 0.1026 |
| VCAM1 | 23.0300 | 13.5600 | 10.4600 | 6.1490 | 0.0350 |
| PECAM1 | 32.9300 | 24.3700 | 22.6500 | 16.6400 | 0.5350 |
| P-selectin | 4.0030 | 8.2800 | 0.4171 | 0.4927 | 0.1952 |
| E-selectin | 3.7230 | 3.4650 | 5.5640 | 10.3400 | 0.7104 |
| EPCR | 50.0700 | 13.4700 | 43.9600 | 8.7160 | >0.9999 |
| NCAM1 | 1.6210 | 2.4580 | 1.0370 | 1.0240 | 0.9709 |
| Isotype | 0.4200 | 0.3337 | 0.2240 | 0.07925 | 0.1667 |

**Supplementary table 8. Comparison of HRGEC cell populations with surface expression of the receptor candidates of interest between unexposed condition and condition with exposure to *P. knowlesi* culture supernatant.**

| **Receptor candidates** | **Unexposed (%)** | | **A1-H.1 (%)** | | **Mann-Whitney P** |
| --- | --- | --- | --- | --- | --- |
|  | **Mean** | **S.D** | **Mean** | **S.D** |  |
| Syndecan 1 | 35.8200 | 7.2210 | 34.3400 | 7.3660 | 0.8413 |
| Syndecan 2 | 5.7840 | 3.1540 | 4.9600 | 2.9830 | >0.9999 |
| CSPG4 | 3.9200 | 4.3160 | 4.0940 | 3.6260 | >0.9999 |
| CD36 | 6.5450 | 5.3590 | 6.9000 | 5.0760 | >0.9999 |
| ICAM1 | 37.6600 | 7.5200 | 39.5800 | 8.8270 | 0.6905 |
| VCAM1 | 25.8200 | 9.7980 | 23.7600 | 4.9160 | >0.9999 |
| PECAM1 | 22.8000 | 8.0260 | 21.1200 | 8.0240 | >0.9999 |
| P-selectin | 2.2520 | 1.1150 | 5.0860 | 4.2570 | 0.4206 |
| E-selectin | 6.4540 | 4.7380 | 6.1260 | 4.6760 | 0.8413 |
| EPCR | 44.6200 | 10.4600 | 44.9800 | 6.7370 | >0.9999 |
| NCAM1 | 5.3520 | 2.0700 | 4.9960 | 3.1960 | 0.6905 |
| Isotype | 1.9680 | 1.7190 | 2.6340 | 3.0480 | >0.9999 |

**Supplementary table 9. MFI of the proteins expressed on hCMEC/D3 under different experimental conditions.**

| **Receptor candidates** | **Unexposed (Arb. units)** | | **A1-H.1 (Arb. units)** | | **Mann-Whitney P** |
| --- | --- | --- | --- | --- | --- |
|  | **Mean** | **S.D** | **Mean** | **S.D** |  |
| Syndecan 1 | 3644.0000 | 1143.0000 | 3111.0000 | 602.0000 | 0.1282 |
| Syndecan 2 | 2077.0000 | 479.6000 | 2201.0000 | 598.7000 | 0.8048 |
| CSPG4 | 1466.0000 | 235.9000 | 1674.0000 | 372.6000 | 0.2593 |
| CD36 | 1284.0000 | 139.7000 | 1568.0000 | 519.2000 | 0.1177 |
| ICAM1 | 9396.0000 | 6491.0000 | 7492.0000 | 4078000 | 0.8048 |
| VCAM1 | 2228.0000 | 630.0000 | 1654.0000 | 617.8000 | 0.0973 |
| PECAM1 | 2068.0000 | 788.6000 | 1817.0000 | 503.1000 | 0.8048 |
| P-selectin | 1406.0000 | 150.6000 | 1702.0000 | 149.9000 | 0.0070 |
| E-selectin | 1356.0000 | 224.4000 | 1787.0000 | 346.3000 | 0.0286 |
| EPCR | 2752.0000 | 773.5000 | 2437.0000 | 783.9000 | 0.7348 |
| NCAM1 | 1867.0000 | 736.9000 | 2097.0000 | 582.8000 | 0.5350 |
| Isotype | 1541.0000 | 231.0000 | 1420.0000 | 502.8000 | 0.1649 |

**Supplementary table 10. MFI of the proteins expressed on HPMEC under different experimental conditions.**

| **Receptor candidates** | **Unexposed (Arb. units)** | | **A1-H.1 (Arb. units)** | | **Mann-Whitney P** |
| --- | --- | --- | --- | --- | --- |
|  | **Mean** | **S.D** | **Mean** | **S.D** |  |
| Syndecan 1 | 2404.0000 | 1571.0000 | 1858.0000 | 253.2000 | 0.7104 |
| Syndecan 2 | 2386.0000 | 1211.0000 | 2220.0000 | 810.7000 | 0.9015 |
| CSPG4 | 2937.0000 | 2261.0000 | 2899.0000 | 1664.0000 | 0.7104 |
| CD36 | 1893.0000 | 642.9000 | 1843.0000 | 233.1000 | 0.1195 |
| ICAM1 | 3317.0000 | 1214.0000 | 3443.0000 | 1156.0000 | 0.8048 |
| VCAM1 | 1724.0000 | 379.1000 | 1608.0000 | 220.5000 | 0.6200 |
| PECAM1 | 10987.0000 | 11792.0000 | 8630.0000 | 7172.0000 | 0.9015 |
| P-selectin | 1734.0000 | 187.3000 | 1904.0000 | 380.2000 | 0.7104 |
| E-selectin | 2131.0000 | 672.2000 | 2433.0000 | 1039.0000 | 0.4557 |
| EPCR | 5303.0000 | 3990.0000 | 6067.0000 | 4631.0000 | 0.4557 |
| NCAM1 | 1702.0000 | 220.8000 | 1722.0000 | 149.0000 | 0.9015 |
| Isotype | 2384.0000 | 575.3000 | 2620.0000 | 331.4000 | 0.3413 |

**Supplementary table 11. MFI of the proteins expressed on HRGEC under different experimental conditions.**

| **Receptor candidates** | **Unexposed (Arb. units)** | | **A1-H.1 (Arb. units)** | | **Mann-Whitney P** |
| --- | --- | --- | --- | --- | --- |
|  | **Mean** | **S.D** | **Mean** | **S.D** |  |
| Syndecan 1 | 2248.0000 | 703.2000 | 1950.0000 | 613.3000 | 0.4206 |
| Syndecan 2 | 1069.0000 | 67.8400 | 1175.0000 | 139.4000 | 0.0794 |
| CSPG4 | 1082.0000 | 53.3200 | 1167.0000 | 69.7300 | 0.0476 |
| CD36 | 1041.0000 | 89.5500 | 1126.0000 | 16.0900 | 0.0556 |
| ICAM1 | 1884.0000 | 386.1000 | 2211.0000 | 496.7000 | 0.5476 |
| VCAM1 | 1286.0000 | 162.6000 | 1349.0000 | 49.5300 | 0.6905 |
| PECAM1 | 1218.0000 | 219.2000 | 1246.0000 | 112.9000 | >0.9999 |
| P-selectin | 1040.0000 | 56.2100 | 1148.0000 | 110.9000 | 0.1825 |
| E-selectin | 1134.0000 | 48.0800 | 1225.0000 | 72.4000 | 0.0317 |
| EPCR | 4515.0000 | 2201.0000 | 5248.0000 | 3627.0000 | >0.9999 |
| NCAM1 | 1248.0000 | 275.6000 | 1279.0000 | 195.4000 | 0.8413 |
| Isotype | 1110.0000 | 87.1800 | 1175.0000 | 87.2000 | 0.2222 |

**Supplementary Figures**


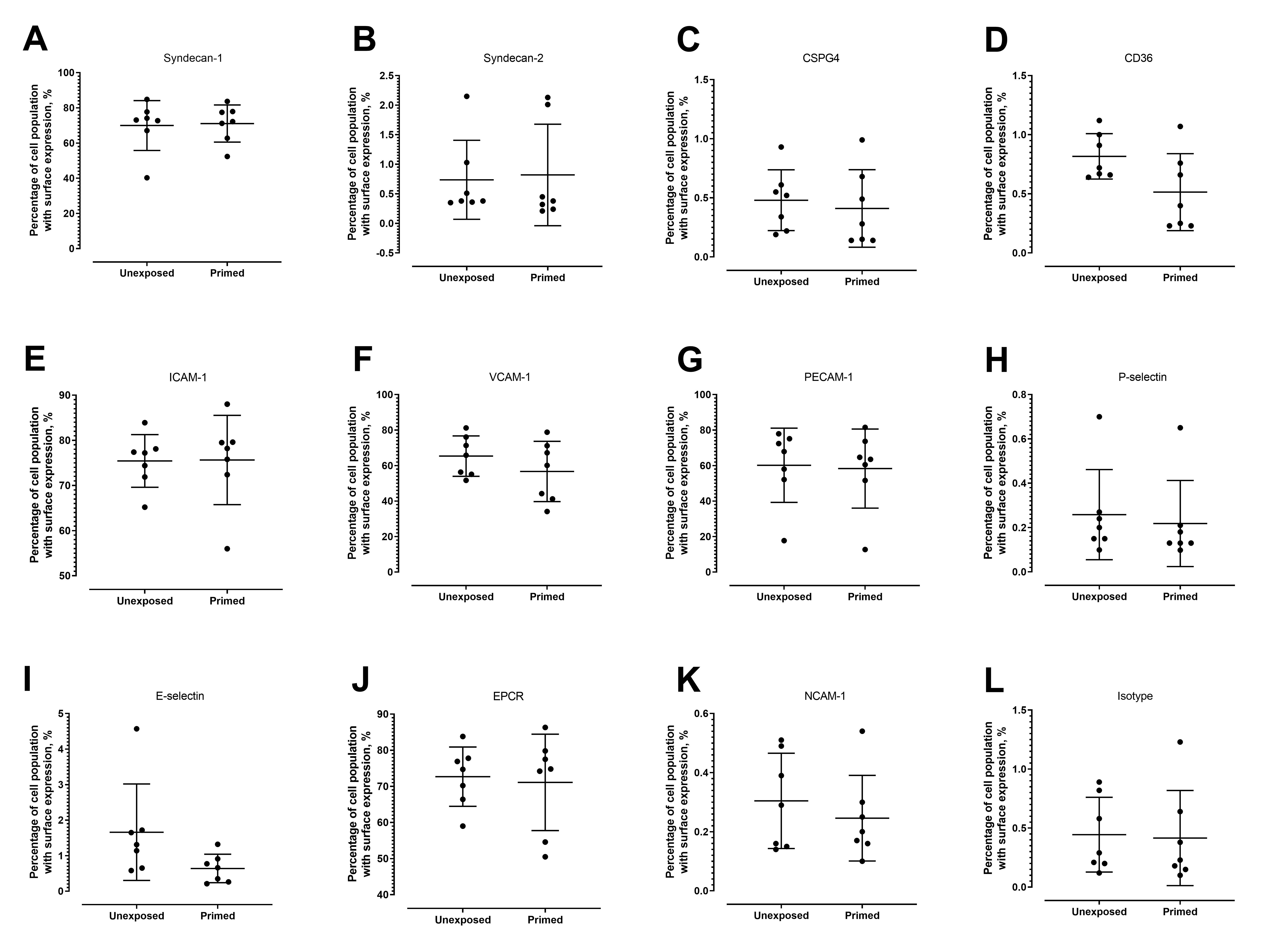


**Supplementary figure 1.** **Percentage of hCMEC/D3 cells with- and without exposure to *P. knowlesi* A1-H.1 expressing protein of interest.** Seven biological replicates were conducted. Based on Mann-Whitney test, no significant difference in expression of the 11 proteins of interest with and without exposure to the parasites.


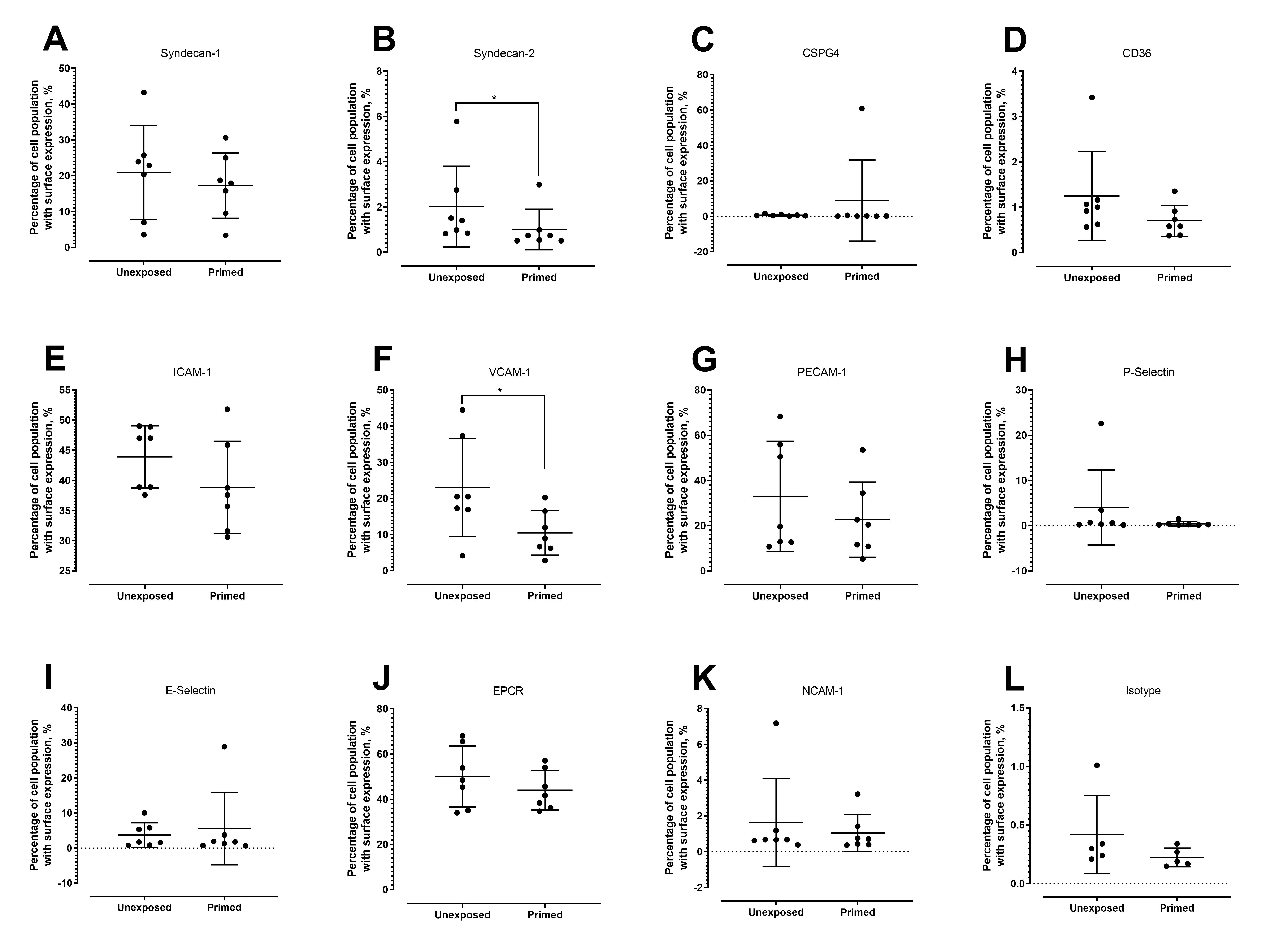


**Supplementary figure 2. Percentage of HPMEC cells with- and without exposure to *P. knowlesi* A1-H.1 expressing protein of interest.** Seven biological replicates were conducted (except for isotype group, which was of five replicates). Based on Mann-Whitney test, cells expressing SDC-2 and VCAM-1 were significantly lower in the exposed group than the unexposed group (P = 0.0466 and 0.035 respectively).


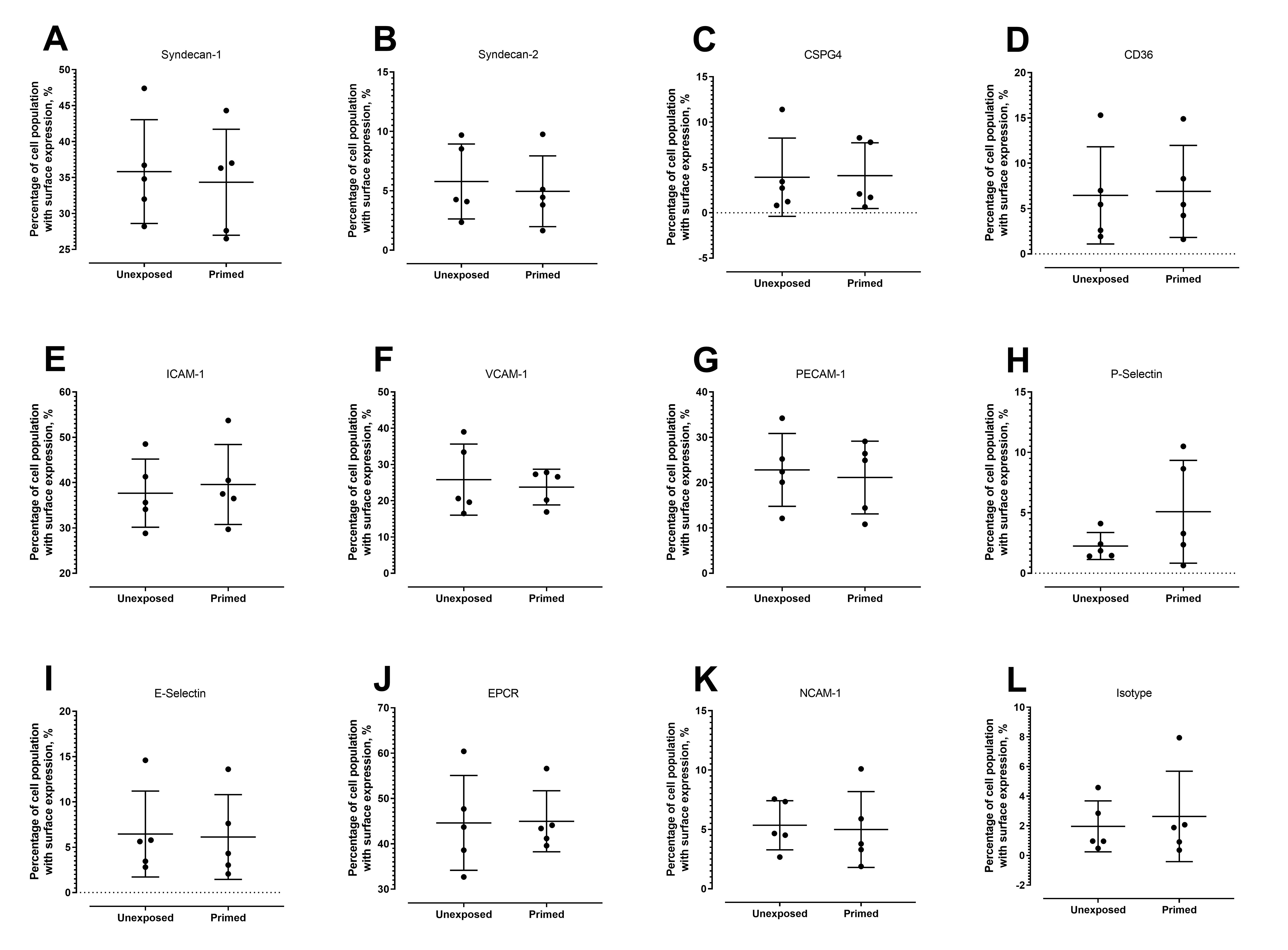


**Supplementary figure 3. Percentage of HRGEC cells with- and without exposure to *P. knowlesi* A1-H.1 expressing protein of interest.** Five biological replicates were conducted. Based on Mann-Whitney test, no significant difference in expression of the 11 proteins of interest with and without exposure to the parasites.
